# Optimization of Extract Method for *Cynomorium songaricum* Rupr. by Response Surface Methodology

**DOI:** 10.1155/2017/6153802

**Published:** 2017-12-24

**Authors:** Xiang Zhang, Caimei Gu, Bashir Ahmad, Linfang Huang

**Affiliations:** ^1^Institute of Medicinal Plant Development, Chinese Academy of Medical Sciences (CAMS), Peking Union Medical College (PUMC), Beijing 100193, China; ^2^Centre for Biotechnology and Microbiology, University of Peshawar, Peshawar, Pakistan

## Abstract

The present study aimed to evaluate the quality of *Cynomorium songaricum* Rupr. from different producing areas, which is an edible, holoparasitic, and desert plant that has been used in traditional medicine for improving immunity and kidney function and treating constipation. We optimized the extract conditions by response surface methodology (RSM) and determined the content of gallic acid, protocatechuic acid, and catechin of *C. songaricum* simultaneously from different producing areas by high-performance liquid chromatography (HPLC). It is the first study in which the RSM was used to optimize the extract condition of *C. songaricum* with multiple evaluation factors, ensuring the efficiency and accuracy of the study. The results were analyzed by principal component analysis, and they showed that the quality of *C. songaricum* from Qinghai Province was the best, while the quality of *C. songaricum* from Gansu Province was the worst. For the first time, the two ecotypes of *C. songaricum*, including Outside Great Wall type and Inside Great Wall type, were discovered and identified by the chemical marker protocatechuic acid. This study is the scientific basis for quality evaluation, especially for food safety.

## 1. Introduction

*Cynomorium songaricum* Rupr. (*C. songaricum*) [1], called Suo Yang in China, is a perennial, holoparasitic, and desert plant, which is spread over the Mediterranean Basin countries, China, and other Central Asian countries [2]. *C. songaricum* is known by different names, the most common being Maltese mushroom, fungus melitensis, champignon or éponge de Malt, fungo di Malta, and tarthuth in Arabic countries [3]. The reason that why it is called mushroom, even if it is a plant, is maybe because of its appearance, the lack of chlorophyll, and the fact that it grows underground. In China, it is a wild plant grown in the Gobi Desert of Inner Mongolia, Gansu Province, Ningxia Province, Xinjiang Province, and Qinghai Province. The roots and fleshy stems of *C. songaricum* have been used as traditional medicine for improving immunity and kidney function and treating constipation [4]. In recent years, the plant extracts have been reported to exhibit antiaging, antidiabetes, scavenging free radicals, anticancer, and so on [5]. It also has been widely used as nutraceuticals for centuries due to its taste, strong tonic effects, and nontoxicity. Moreover, the extracts and preparations of *C. songaricum* for preventing skin aging, stimulating the growth of hair, and treating erectile dysfunction are in the United States and Japan [6]. In the previous work, we have revealed the ecological variation, ecotype differentiation, and formation of traditional Chinese medicinal materials, and we applied them in American ginseng [7, 8] and made the regional suitability evaluation on desert Herb Cynomorii [9].

Without a doubt, the good effects are closely related to the chemical components. Previous studies have shown that numerous chemical components exist in *C. songaricum* including condensed tannins, steroids, triterpenes, butyl fructosides, flavonoids, lignin glycosides, and alkaloids. Among all the components, gallic acid, protocatechuic acid, and catechin are the main active ingredients [10]. Many factors such as material-to-solvent ratio, solvent composition, extraction time, and ultrasonic power may significantly influence the extraction efficacy. Generally, optimization of a process could be achieved by either empirical or statistical methods, but the empirical methods have limitations toward complete optimization. The traditional one-factor-at-a-time method spent a lot of time to process optimization. In addition, the interactions among different factors may be ignored which results in the chance of approaching a real optimum which is very impossible [11–13].

RSM, originally described by Box and Wilson, is a statistical mathematical method, which uses a minimum of resources and quantitative data from an appropriate experimental design to determine, and simultaneously solve, multivariate equations for optimizing process or products [14, 15]. RSM is an effective tool for developing, improving, and optimizing the processes when many factors and interactions affect the desired response. RSM has been successfully applied to model and optimize biochemical and biotechnological processes related to food systems [16, 17].

Previous studies have investigated the orthogonal test applied to optimize the *C. songaricum* extraction [18, 19]. But the RSM has not been used in optimizing the extraction of gallic acid, protocatechuic acid, and catechin of *C. songaricum*. In this study, optimization of experimental conditions that result in the highest content determination of gallic acid, protocatechuic acid, and catechin exracts was conducted for the first time. Additionally, different producing areas of *C. songaricum* were identified and evaluated by the optimized extraction conditions.

## 2. Materials and Methods

### 2.1. Reagents, Chemicals, and Samples

**Figure 1:**
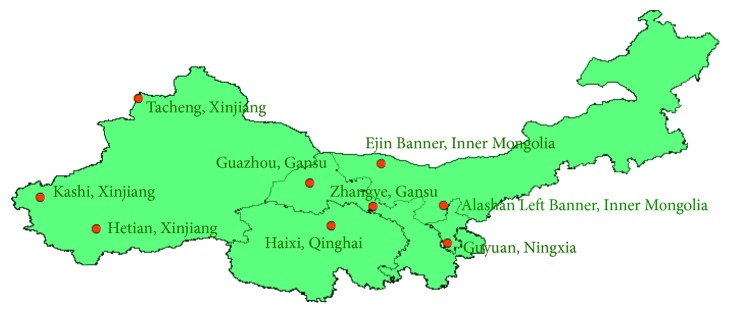
The sampling points of *C. songaricum*.

The standard compounds of gallic acid, protocatechuic acid, and catechin were purchased from Chengdu Must Bio-Tech. Co., Ltd. (Chengdu, China). *C. songaricum* samples were collected from Gansu, Qinghai, Xinjiang, Ningxia, and Inner Mongolia (China) in 2015 and 2016 (Figure 1). The samples were identified by Professor Linfang Huang in the Institute of Medicinal Plant Development, Chinese Academy of Medical Sciences and Peking Union Medical College, Beijing, China. Voucher specimens were stored in the resource center of this institution.  Table 1 shows the sample list. The phosphoric acid used in this study was of analytical grade (purchased from Tianjin Guangfu Fine Chemical Industry Research Institute). The methanol and acetonitrile were of chromatographic grade and purchased from Thermo Fisher Scientific (China) Co., Ltd. The purified water purchased from Hangzhou Wahaha Group Co., Ltd. was used for the preparation of solutions.

### 2.2. Instruments

A Waters Breeze series LC system purchased from Waters Technology (Shanghai) Co., Ltd., equipped with a 1525 binary pump, a 2487 dual λ absorbance detector performing the wavelength of 260 nm, a 717 plus autosampler, and a Breeze Chemstation software, was used in the novel HPLC-UV method. The injection volumes were 10 μL for each run. The ultrasonic used in the study was purchased from Kunshan Ultrasonic Instruments Co., Ltd. (KQ-400KDE). The centrifuge was purchased from Germany (Sigma).

### 2.3. Sampling and Pretreatment

Samples were grounded into powder using an electric grinder for 5 min and then passed through a 50-mesh sieve. One gram of sample was extracted with methanol (20 mL) by the methods of ultrasonication extraction (80 w, 40 min), standing for 10 min, centrifuging (8000 r, 5 min), and then the supernatant was filtered. The extracts were kept at 4°C in the refrigerator for further experiments.

### 2.4. Standard Preparation

Accurately, 4.33 mg gallic acid, 5.32 mg protocatechuic acid, and 4.48 mg catechin were weighed and dissolved in 2 mL of methanol. Appropriate dilutions were made to give a stock standard concentration of 0.2 mg/mL, 0.15 mg/mL, and 0.1 mg/mL for gallic acid, protocatechuic acid, and catechin, respectively. Concentrations were calculated from peak area determined by manual integration.

### 2.5. High-Performance Liquid Chromatography-UV

Chromatographic separation of methanol extracts (10 μL) was conducted using a C18, reverse-phase (5 μm), Atlantis T3 column (250 × 4.6 mm I.D.; Waters, USA). The mobile phase consisted of 0.02% phosphoric acid in water (solvent B) and acetonitrile (solvent A) with the following gradients: 10% A for 5 min, to 13% A in 21 min, to 30% A in 4 min, and to 40% A in 5 min. The flow rate was 1 ml/min, the temperature was 30°C, and injection volume was 10 μL.

### 2.6. Selection of Appropriate Extraction Conditions

The initial step of the preliminary experiment was to select appropriate extraction conditions for gallic acid, protocatechuic acid, and catechin. The three compounds from *C. songaricum* were extracted using methanol varying in the range of 60–100% (v/v; water/methanol). The second step of the preliminary experiment was to determine the extraction material ratio. The three compounds from *C. songaricum* were extracted using the best solvent composition chosen in the previous step. The ultrasonic time varied from 20 to 60 min while holding the ultrasonic power course constant at 80 W. The final step of the preliminary experiment was to select the appropriate ultrasonic power course for the extraction of gallic acid, protocatechuic acid, and catechin.

### 2.7. Box-Behnken Design

According to the principle of the Box-Behnken design, material-to-solvent ratio, ultrasonic time, and ultrasonic power were identified to have strong effects on the response in preliminary one-factor-at-a-time experiments. Three-factor, 3-level Box-Behnken design was used. A total of 17 experiment runs with five center points were generated by design expert software, and the total number of experiments (*N*) was calculated as follows: *N* = *K* ^2^ + *K* + *C*_*p*_, where *K* is the factor number and *C*_*p*_ is the replicate number of the central point. The nonlinear computer generated quadratic model is shown in the following:

(1) $$\begin{array}{c} R=b_{0}+b_{1}X_{1}+b_{2}X_{2}+b_{3}X_{3}+b_{12}X_{1}X_{2}+b_{13}X_{1}X_{3}+b_{23}X_{2}X_{3}+b_{11}X_{1}^{2}+b_{22}X_{2}^{2}+b_{33}X_{3}^{3}. \end{array}$$

The response was the measurement of each factor level combination. *b*_0_ is the intercept; *b*_1_, *b*_2_, and *b*_3_ are the linear coefficients; *b*_12_, *b*_23_, and *b*_13_ are the interaction coefficients, while *b*_11_, *b*_22_, and *b*_33_ are the quadratic coefficients. The independent variables were material-to-solvent ratio (*X*_1_), ultrasonic time (*X*_2_), and ultrasonic power (*X*_3_) while the content of gallic acid (*Y*_1_), protocatechuic acid (*Y*_2_), and catechin (*Y*_3_) were the dependent variables [20, 21]. The range of independent variables was chosen on the basis of the result of various initial trials. Here, all the variables including material-to-solvent ratio, ultrasonic time, and ultrasonic power were studied at three different levels: low (−1), medium (0), and high (+1). Various formulations were prepared according to the independent variables selected as shown in Table 2.

### 2.8. Content Determination and Data Normalization

We determined the content of *C. songaricum* for 15 groups, experiment using the HPLC method discussed earlier. In order to comprehensively reflect the results, the three evaluation indicators are normalized to one indicator (*D*) by the normalization method of the desirability function approach. The calculating formula is shown in the following:

(2) $$\begin{array}{c} d_{i}=\frac{\left(Y_{i}-Y_{\min}\right)}{\left(Y_{\max}-Y_{\min}\right)}\;,D={\left(d_{1}\times d_{2}\times ...\times d_{i}\right)}^{1/i}. \end{array}$$

### 2.9. Statistical Analysis

Multiple regression analysis and Pareto analysis of variance (ANOVA) were conducted for fitting the mathematical model using Design-Expert software (Version 8.0.5b). The modeling was started with a quadratic model. Significant terms in the model for each response were found by analysis of variance, and the significance was judged by the F-statistic calculated from the data. The data of the experiment were evaluated with various descriptive statistical analyses to reflect the statistical significance of the developed quadratic mathematical model. After fitting the data to the models, the generated data were used for plotting response surface and contour plots [22].

#### 2.9.1. Sample Identification and Analysis

Additionally, the samples of *C. songaricum* from different regions were identified by the optimal experimental conditions, which were obtained from the response surface methodology experiment. The results were analyzed through principal component analysis (PCA).

## 3. Results and Discussion

### 3.1. Method Validation

The method exhibited a good linearity of gallic acid, protocatechuic acid, and catechin which was described in Table 3.

### 3.2. Box-Behnken Design Analysis

In order to study the combined effect of independent variables (material-to-solvent ratio, ultrasonic time, and ultrasonic power) on the extraction, experiments were performed for different combinations of parameters using statistically designed experiments, and results are shown in Table 4, which includes the design and the experimental and predicted values.

### 3.3. Effect of Process Variables on the Content Determination of C. songaricum

**Figure 2:**
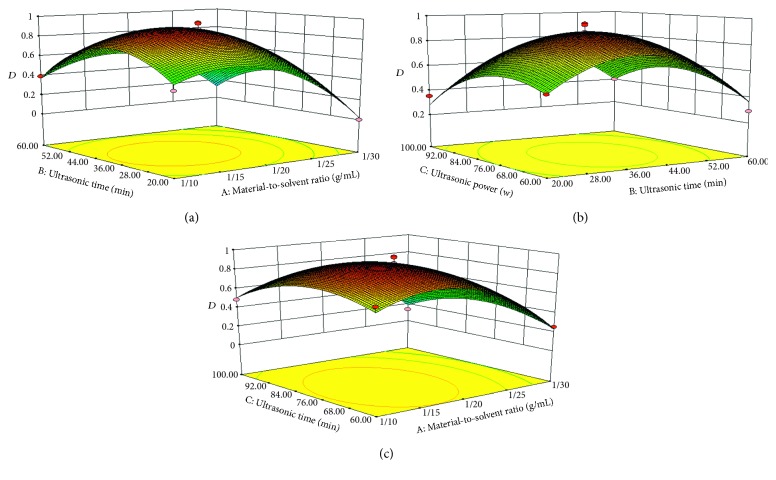
(a) 3D graphic surface optimization of *D* versus ultrasonic time and material-to-solvent ratio; (b) 3D graphic surface optimization of *D* versus ultrasonic power and ultrasonic time; (c) 3D graphic surface optimization of *D* versus ultrasonic power and material-to-solvent ratio.

Three factors at three-level Box-Behnken design were used in this study to investigate the influence of process variables such as material-to-solvent ratio, ultrasonic time, and ultrasonic power. From the developed model, the three-dimensional response surface and contour plots were constructed to illustrate the main and interactive effects of independent variables on a response variable. The figures are drawn by a two-factor constant, whereas the other two factors are varied in order to understand their main and interactive effects on dependent variables. It is also used to locate the optimum conditions. From Figure 2, it is clear that *D* increase with the conditions including increasing ultrasonic time ranges from 20 to 40 (min), increasing material-to-solvent ratio ranges from 1 : 10 to 1 : 17 (g/mL), and increasing ultrasonic power ranges from 60 to 76 (W). But slowly decreases when the conditions are out of the ranges. Derringer's desired function methodology was employed to optimize the extraction process conditions on the maximum extractive capacity of gallic acid, protocatechuic acid, and catechin from *C. songaricum* as follows: material-to-solvent ratio of 1 : 17, ultrasonic time of 39 min, and ultrasonic power of 76 W.

A desirability ramp was developed from optimal points via a numerical optimization technique. For the validation of the optimum conditions, triplicate confirmatory experiments were carried out under the optimized conditions. All results are closely related to the data obtained from optimization analysis, which indicate that the Box-Behnken design could be effectively used to optimize the extraction parameters.

### 3.4. Content Analysis of Different Components from Various Origins

**Figure 3:**
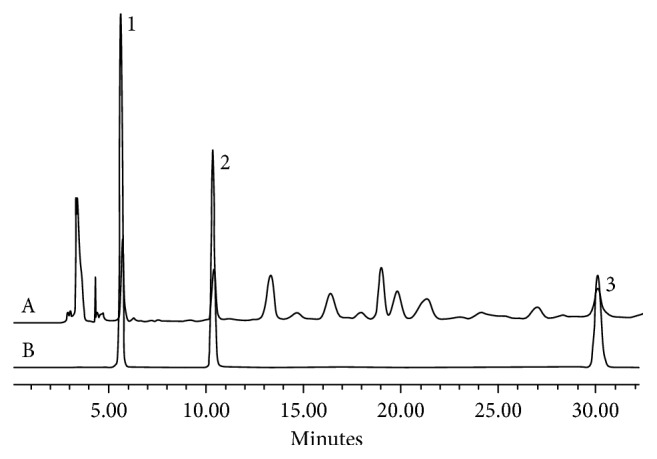
The HPLC chromatogram of *C. songaricum*; A: crude drugs; B: reference standard; 1: gallic acid; 2: protocatechuic acid; and 3: catechin.

Figure 3 shows the chromatograms of the sample (Figure 3) and the reference standard (Figure 3). In the chromatogram conditions, the main peaks and the nearby peaks were separated well, and the running time was within 31 minutes, which revealed that the content determination method of *C. songaricum* we built was accurate and efficient. The chromatogram conditions can be used to analyze the samples of *C. songaricum* from different origins.

**Figure 4:**
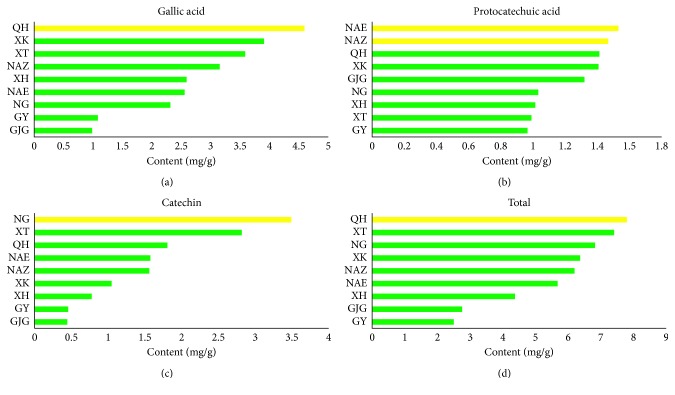
Comparison of content determination of *C. songaricum* between different producing areas from China.

The data of content determination were shown in Figure 4 (the specific concentration of three compounds can be seen in see Supplemental Table S1 available online at https://doi.org/10.1155/2017/6153802). The samples from QH contained the highest content of gallic acid and total three components (Figures 4(a)(a) and 4(d)(d)), and the samples from Inner Mongolia had the highest content of protocatechuic acid (Figure 4(b)(b)). In Figure 4(c)(c), the catechin was determined the highest in the samples from NG, followed by XT samples. According to the comprehensive analysis of the content data in this study, the quality of *C. songaricum* from QH was the best, while the samples from Gansu were the worst.

**Figure 5:**
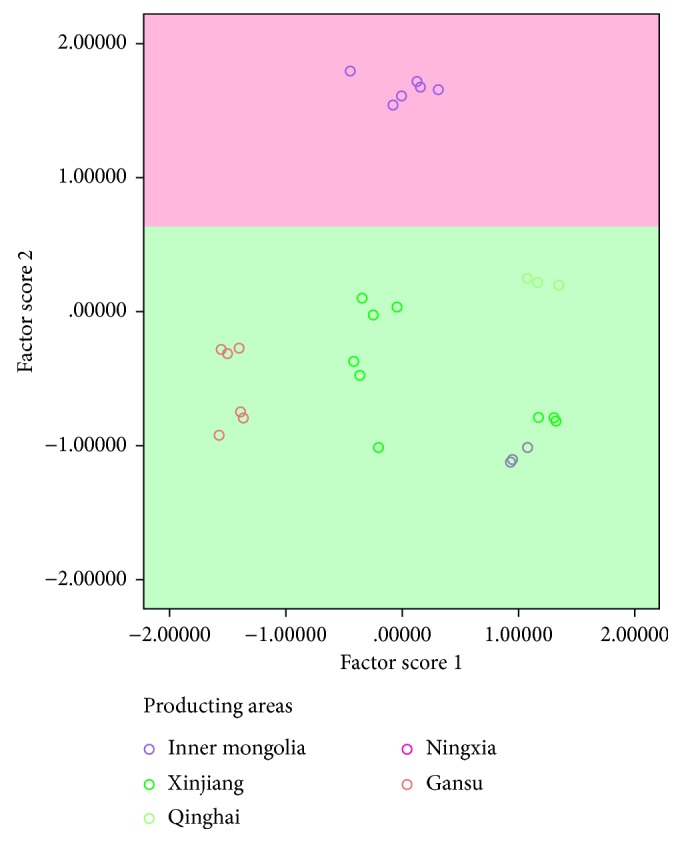
The score plot of PCA of *C. songaricum*.

Additionally, the content data were analyzed by PCA (Figure 5). The results showed that the samples from Inner Mongolia could be separated from other samples, which might be for the reason that they located in different geographical positions. The Great Wall and its extended boundary lines could divide all the samples into two parts, namely, Outside Great Wall and Inside Great Wall. Inner Mongolia located in the outside of the Great Wall while the other provinces came from the inside of the Great Wall. Therefore, there were two ecotypes about *C. songaricum*, including Outside Great Wall type and Inside Great Wall type. From the analysis of Figures 4 and 5, we can conclude that the difference between Inner Mongolia and other origins was the content of protocatechuic acid. Only the samples from Inner Mongolia contained the obviously higher content of protocatechuic acid than others, which could be the chemical marker to identify the different ecotypes of *C. songaricum*. All the samples of Xinjiang clustered to one group except XT, which grouped together with NG. On the view of chemical components, the higher content of gallic and catechin in the sample of XT and NG resulted in them being near each other in Figure 5.

## 4. Conclusions

It is the first study in which the RSM was used to optimize the extract condition of *C. songaricum* with multiple evaluation factors, ensuring the efficiency and accuracy of the study. In this study, the quality of *C. songaricum* from QH was the best, while Gansu was the worst. For the first time, we divided *C. songaricum* into two ecotypes, including the Outside Great Wall type and Inside Great Wall type. *C. songaricum* from Inner Mongolia was the Outside Great Wall type, and other origins were the Inside Great Wall type. In addition, the chemical marker was protocatechuic acid. In the future, we will focus on finding the reason at molecular level why Inner Monngolia is different from XT. Our study is important to the clinical practice and industry because *C. songaricum* from QH with the highest quality can be the first choice for application. This study is the scientific basis for quality evaluation, and it provides the research direction for the future.

## Supplementary Material

Table S1: The specific concentration of gallic acid, protocatechuic acid, and catechinic acid in C.songaricum

## Figures and Tables

**Table 1:** The sample list of *C. songaricum*.

Number	Name	Origin	Time
1	NAE-1	Ejin Banner, Inner Mongolia, China	May, 2015
2	NAE-2	Ejin Banner, Inner Mongolia, China	May, 2015
3	NAE-3	Ejin Banner, Inner Mongolia, China	May, 2015
4	NAZ-1	Alashan Left Banner, Inner Mongolia, China	May, 2015
5	NAZ-2	Alashan Left Banner, Inner Mongolia, China	May, 2015
6	NAZ-3	Alashan Left Banner, Inner Mongolia, China	May, 2015
7	XH-1	Hetian, Xinjiang, China	May, 2016
8	XH-2	Hetian, Xinjiang, China	May, 2016
9	XH-3	Hetian, Xinjiang, China	May, 2016
10	XT-1	Tacheng, Xinjiang, China	May, 2016
11	XT-2	Tacheng, Xinjiang, China	May, 2016
12	XT-3	Tacheng, Xinjiang, China	May, 2016
13	XK-1	Kashi, Xinjiang, China	May, 2016
14	XK-2	Kashi, Xinjiang, China	May, 2016
15	XK-3	Kashi, Xinjiang, China	May, 2016
16	QH-1	Haixi, Qinghai, China	May, 2015
17	QH-2	Haixi, Qinghai, China	May, 2015
18	QH-3	Haixi, Qinghai, China	May, 2015
19	NG-1	Guyuan, Ningxia, China	May, 2015
20	NG-2	Guyuan, Ningxia, China	May, 2015
21	NG-3	Guyuan, Ningxia, China	May, 2015
22	GJG-1	Guazhou, Gansu, China	May, 2015
23	GJG-2	Guazhou, Gansu, China	May, 2015
24	GJG-3	Guazhou, Gansu, China	May, 2015
25	GY-1	Zhangye, Gansu, China	May, 2015
26	GY-2	Zhangye, Gansu, China	May, 2015
27	GY-3	Zhangye, Gansu, China	May, 2015

**Table 2:** Independent and dependent variables used in the Box-Behnken design for the optimization of *C. songaricum* extraction.

Factor	Level used, actual coded		
Independent variables	Low (−1)	Medium (0)	High (+1)
*X*_1_ = material-to-solvent ratio (g/mL)	1/10	1/20	1/30
*X*_2_ = ultrasonic time (min)	20	40	60
*X*_3_ = ultrasonic power (W)	60	80	100

Dependent variables			Goal

*Y*_1_ = content of gallic acid			Maximize
*Y*_2_ = content of protocatechuic acid			Maximize
*Y*_3_ = content of catechin			Maximize

**Table 3:** The parameter optimization of method validation.

Compounds	Concentrations (mg/L)	Linear equations	*R* ^2^	RSD of interday and intraday	RSD of stability
Gallic acid	0.04–0.4	*y* = 1995100.0000*x* + 48792.0000	0.9992	0.36	1.1
Protocatechuic acid	0.03–0.3	*y* = 286428.3894*x* + 3169.2192	0.9992	0.57	2.0
Catechin	0.02–0.2	*y* = 2973976.1835*x* + 8138.8420	0.9994	0.31	1.9

**Table 4:** Observed response in the Box-Behnken design for optimization of *C. songaricum* extraction formulations with predicted values generated by Design-Expert software (three variables: *X*_1_: material-to-solvent ratio, *X*_2_: ultrasonic time, and *X*_3_: ultrasonic power. *D*: desirability function).

Formulations	Independent variables			Actual value	Predicted value
	*X*_1_	*X*_2_	*X*_3_	*D*	*D*
1	1/10	20	80	0.46	0.50
2	1/30	20	80	0.00	0.02
3	1/10	60	80	0.39	0.37
4	1/30	60	80	0.12	0.078
5	1/10	40	60	0.71	0.66
6	1/30	40	60	0.25	0.23
7	1/10	40	100	0.49	0.51
8	1/30	40	100	0.14	0.18
9	1/20	20	60	0.52	0.53
10	1/20	60	60	0.29	0.35
11	1/20	20	100	0.35	0.29
12	1/20	60	100	0.40	0.40
13	1/20	40	80	0.95	0.89
14	1/20	40	80	0.88	0.89
15	1/20	40	80	0.87	0.89
16	1/20	40	80	0.81	0.89
17	1/20	40	80	0.94	0.89

## Abbreviations

HPLC: High-performance liquid chromatography
RSM: Response surface methodology
PCA: Principal component analysis
XT: Tacheng, Xinjiang Province
QH: Haixi, Qinghai Province
NG: Guyuan, Ningxia Province.
