# Supplementary material for: Optimization of Extract Method for Cynomorium songaricum Rupr. by Response Surface Methodology
Source: J Anal Methods Chem. 2017 Dec 24;2017:6153802. doi: 10.1155/2017/6153802 (PMC5757168; doi:10.1155/2017/6153802)
Supplement: Supplementary file 1 — Table S1: The specific concentration of gallic acid, protocatechuic acid, and catechinic acid in C.songaricum [file 6153802.f1.pdf]

Table S1 The specific concentration of gallic acid, protocatechuic acid and catechinic acid

Name	Origins	Concentration of protocatechuic acid(mg/g)	Concentration of gallic acid(mg/g)	Concentration of catechinic acid(mg/g)
NAE-1	Ejin Banner, Inner Mongolia, China	2.55272	2.86688	1.86712
NAE-2	Ejin Banner, Inner Mongolia, China	2.59224	2.70930	1.69581
NAE-3	Ejin Banner, Inner Mongolia, China	2.55702	2.56103	0.83573
NAZ-1	Alashan Left Banner, Inner Mongolia, China	1.42056	3.32633	1.82363
NAZ-2	Alashan Left Banner, Inner Mongolia, China	1.43432	3.00155	1.52200
NAZ-3	Alashan Left Banner, Inner Mongolia, China	1.54541	3.12402	1.35943
XH-1	Hetian, Xinjiang, China	1.16629	2.62403	0.78713
XH-2	Hetian, Xinjiang, China	0.74675	2.61937	1.14990
XH-3	Hetian, Xinjiang, China	1.12079	2.53212	0.39335
XT-1	Tacheng, Xinjiang, China	1.00659	3.68099	2.80528
XT-2	Tacheng, Xinjiang, China	0.97941	3.57341	2.67895
XT-3	Tacheng, Xinjiang, China	0.98018	3.52356	2.98032
XK-1	Kashi, Xinjiang, China	1.35155	4.03247	1.00608
XK-2	Tacheng, Xinjiang, China	1.40580	3.91257	1.18352
XK-3	Tacheng, Xinjiang, China	1.46745	3.77348	0.95283
QH-1	Haixi, Qinghai, China	1.39806	4.56203	1.78351
QH-2	Haixi, Qinghai, China	1.43219	4.48416	1.69971
QH-3	Haixi, Qinghai, China	1.41351	4.74657	1.93041
NG-1	Guyuan, Ningxia, China	1.07798	2.39748	3.57698
NG-2	Guyuan, Ningxia, China	0.99924	2.28950	3.42690

NG-3	Guyuan, Ningxia, China	1.02317	2.25548	3.48367
GJG-1	Guazhou, Gansu, China	1.32522	0.88451	0.39321
GJG-2	Guazhou, Gansu, China	1.29298	0.98505	0.39730
GJG-3	Guazhou, Gansu, China	1.33288	1.02836	0.52992
GY-1	Zhangye, Gansu, China	0.96658	1.17444	0.44799
GY-2	Zhangye, Gansu, China	0.98475	1.19542	0.39181
GY-3	Zhangye, Gansu, China	0.94647	0.68712	0.52948
